# The Effect of an Educational Booklet on Women’s Knowledge and Intentions to Use Contraception

**DOI:** 10.7759/cureus.71466

**Published:** 2024-10-14

**Authors:** Hamdah M Alruwaili, Hanan A Badr

**Affiliations:** 1 Academic Affairs and Training, Aljouf Health Cluster, Sakaka, SAU; 2 Faculty of Nursing, King Abdulaziz University, Jeddah, SAU

**Keywords:** educational booklet, knowledge, modern contraception, use, women

## Abstract

Background

Contraception has emerged as a highly debated issue globally in recent years. Effectively spacing and limiting pregnancies positively influences women’s health and well-being, as well as the outcomes of each pregnancy. Conversely, unintended pregnancies resulting from the lack of contraceptive use can jeopardize women’s health and place a significant burden on society.

Aim

This study aimed to assess the effect of an educational booklet on the knowledge and intention of primiparae women to use modern contraception.

Methods

The study utilized a quasi-experimental design with pre-test and post-test assessments conducted in Saudi Arabia. A purposive sampling technique was employed to select participants, resulting in a sample size of 60 primiparae women. A self-administered questionnaire was used to evaluate the participants’ knowledge and intention to use contraception both before and after the educational session.

Results

The results demonstrated a significant improvement in participants’ knowledge when comparing assessments before and after the educational session. Initially, 88.3% of participants exhibited poor knowledge, while only 11.7% had an average level of understanding. Immediately following the educational session, 91.7% of participants attained a good level of knowledge, and after one month, 76.7% maintained good knowledge, with 15% reporting an average level. After one month, the most commonly used contraceptive method was oral contraceptive pills (25%), followed by intrauterine devices (21.7%) and contraceptive implants (21.7%). Notably, 70% of participants had not previously used any contraceptive method.

Conclusions

The educational session utilizing an educational booklet significantly enhanced participants’ knowledge and intention to use contraceptives. Consequently, it is crucial for health authorities to address the need for expanding family planning services. Future studies may benefit from employing a mixed-methods research design, incorporating both quantitative and qualitative approaches, to explore barriers to contraceptive use and the challenges of implementing contraceptive education programs in healthcare facilities across Saudi Arabia.

## Introduction

Contraception has become a highly debated issue worldwide in recent years [1]. It refers to the prevention of pregnancy through the deliberate use of techniques, devices, or medications that interfere with natural reproductive processes, such as ovulation or egg implantation. WHO has identified pregnancy spacing as a critical measure for promoting maternal health [2].

Unintended pregnancies due to the lack of contraceptive use can jeopardize women’s health and impose significant societal burdens [1]. Early pregnancies are associated with high perinatal and maternal mortality rates, as well as increased childbirth complications [3]. According to WHO, 60% of maternal deaths occur during the postpartum period, with 45% occurring within the first day of delivery, 65% within the first week, and 80% within the first two weeks after birth [4].

The prevalence of contraceptive use in the Kingdom of Saudi Arabia remains low compared to other countries. A 2020 report by the General Authority for Statistics revealed that a 2018 household health survey found that approximately 33% of married women aged 15-49 years were using contraceptive methods. Additionally, as Saudi society undergoes rapid sociodemographic changes, including an increasing number of women entering the workforce, the need for contraception has become increasingly crucial for women of childbearing age [1].

Spacing and limiting the number of pregnancies positively impact women’s health, well-being, and the outcomes of each pregnancy [5]. Studies conducted in the country have shown that women primarily rely on friends and relatives for information about contraceptive methods, followed by the Internet, health personnel, and books as sources [6].

However, numerous studies indicate that a lack of education and knowledge, along with fears of adverse effects, contribute to the avoidance of contraceptive methods [5]. Additionally, myths and misconceptions about various contraceptive options further discourage or prevent women from utilizing them [3].

Research conducted in Jeddah assessed the awareness of 979 women regarding birth control methods and revealed significant gaps in their understanding. The researchers recommended implementing effective strategies for contraceptive education to dispel misconceptions, enhance awareness, and promote the appropriate use of contraception [7].

In Saudi Arabia and the broader Middle East, studies on contraceptive use and family planning remain limited [7]. Integrating contraception education into obstetric care has the potential to enhance postpartum contraceptive use [8]. Therefore, this paper aims to evaluate the effect of an educational booklet on primiparae women’s knowledge and intention to use contraception in Jeddah, Saudi Arabia. Based on the results, we intend to develop an educational booklet that elucidates contraceptive options, raises awareness, and fosters contraceptive acceptance among vulnerable women.

## Materials and methods

Study design

We employed a quasi-experimental one-group pretest-posttest design to evaluate the impact of an educational booklet on primiparae women’s knowledge and their intention to use contraception.

Setting

We conducted this study in Jeddah, one of the largest cities in Saudi Arabia.

Sample

We utilized a convenience sampling technique to select the study participants and calculated the sample size using the Stephen Thompson formula, in consultation with a statistician (CI = 95%, power = 0.8, confidence limit = 0.05). The final sample consisted of 60 primiparae women.

Inclusion criteria

The inclusion criteria for this study were as follows: participants had to be primiparae, admitted to the postnatal ward, free from any medical or surgical complications, aged 20 to under 35 years, willing to participate, and able to read and write in Arabic.

Study instruments

The study questionnaire comprised four parts. For the assessment of contraception knowledge and contraceptive intent, the researchers obtained permission to use and adapt the tools. A pilot study was conducted with 19 participants, who were not included in the actual study. All 19 participants understood the questions and completed the questionnaires within 14 minutes, encountering no apparent difficulties. The purpose of the pilot study was to evaluate the validity of the instruments and assess the reliability and validity of the data collection process and scales employed.

Part I: Sociodemographic Characteristics

In the first part, we collected participants’ sociodemographic data, including age, education, occupation, income, husband’s education, family residence location, and access to family planning services. Responses were categorical. This section also identified each participant’s source of information about contraception, providing five possible choices.

Part II: Contraception Knowledge Assessment (CKA)

We developed a CKA tool based on a literature review [7], which was validated by three faculty members. The self-reported instrument consisted of 16 items aimed at assessing contraceptive knowledge. We evaluated the internal consistency reliability of the CKA using Cronbach’s alpha, which was found to be 0.89. Participants received 1 point for correct answers and 0 points for incorrect or “I don’t know” responses. Knowledge levels were categorized as poor (under 40%), adequate (40%-60%), or good (above 60%).

Part III: Contraceptive Intent Questionnaire (CIQ)

The Jeddah Office for Certified Translation translated the CIQ, designed and validated by Raine-Bennett and Rocca [9], into Arabic. After modifications for content validity, the CIQ consisted of 15 items: the first explored previously used contraceptive methods, the second covered chosen methods, and the third asked about the reasons for selecting them. The remaining 12 items were rated on a 4-point Likert scale to assess the intent to use contraception, with total scores ranging from 12 to 48. Higher scores indicated a greater intent to use contraception. The reliability of the CIQ was deemed good, with a Cronbach’s alpha of 0.73.

Part IV: Feedback on the Educational Booklet

In the final section, we assessed participants’ feedback on the educational booklet. This section comprised four questions evaluating the booklet’s usefulness, the benefits participants derived from it, and their likelihood of recommending it to others. Responses were recorded as yes (1) or no (0).

Educational booklet

The educational booklet was developed by the researcher and reviewed for content validity by three faculty members. Following their feedback, the content was revised, and the finalized booklet was prepared for implementation and testing. The needs of mothers, along with the selected research objectives, were taken into account when creating the booklet’s content.

Designed to enhance women’s knowledge and use of contraceptive methods, the booklet aimed to correct any misconceptions they might have. It included information on the importance of family planning, various contraceptive methods, notes on utilization, effectiveness, advantages, and disadvantages of each method, contraindications, and a comprehensive index and references. The content was organized in a logical sequence. After conducting the pilot study, an appropriate timeframe for the educational session was established, and the final version of the educational booklet was printed and prepared for use.

Data collection

We consecutively invited patients during daily visits to the obstetrics unit, conducting participant meetings in their private rooms during postpartum care. After explaining the study’s purpose to those who met the inclusion criteria and agreed to participate, we assured them that they could withdraw at any time without penalty. Each participant signed a consent form that outlined the study’s aim and guaranteed anonymity.

Participants initially completed the pre-test using the CKA and CIQ during individual meetings with us. The educational session, which introduced the content of the contraception booklet, lasted 40-45 minutes. Following the session, participants took a post-test and a follow-up test one month later, utilizing the same instruments. Data collection was completed over a span of 16 weeks.

Ethical considerations

We obtained ethical approval for this study from the Institutional Review Board of Health Sciences at King Abdulaziz University Hospital (KAUH) and the Faculty of Nursing Ethics Committees (reference no. 82-20) prior to commencing data collection. Participants also provided informed consent before completing the questionnaires.

Statistical analysis

We utilized IBM SPSS Statistics for Windows, Version 22.0 (Released 2013; IBM Corp., Armonk, NY, USA) for the quantitative data analysis. Descriptive statistics, including demographic summaries, means, and SDs, were employed to summarize the data. To identify differences in knowledge levels and intent to use contraceptives based on participants’ demographic factors, independent sample t-tests and one-way ANOVA were conducted. A two-sided p-value of <0.05 was considered statistically significant. Additionally, a paired t-test was used to evaluate the effect of the educational intervention on participants’ knowledge and intent to use contraceptives.

## Results

The present study involved 60 women who were invited by the researcher to participate.

Sociodemographic characteristics of the participants

In Table 1, 38.3% (N = 23) of the participants were aged 25-30 years, while 36.7% were in the 20-25 age group. Three-quarters of the participants held university degrees, and the remaining quarter completed secondary school. More than half (63.3%, N = 38) reported an income exceeding 6,000 SAR. Additionally, 66.7% (N = 40) of the participants were homemakers. Approximately three-quarters of the participants’ husbands also held university degrees, and a vast majority (95%, N = 57) resided in urban areas.

**Table 1:** Distribution of study participants according to sociodemographic characteristics

Variables	N	%	
Age groups			
20 to <25 years	22	36.7	
25 to <30 years	23	38.3	
30 to <35 years	15	25	
Income			
<3,000 SAR	5	8.3	
3,000-6,000 SAR	17	28.3	
>6,000 SAR	38	63.3	
Employment			
Housewife	40	66.7	
Employee	8	13.3	
Student	12	20	
Education			
Secondary	15	25	
University	45	75	
Husband’s education			
Intermediate and secondary	15		25
University	45		75
Residence			
Urban area	57		95
Rural area	3		5

The results further indicated that 70% (N = 42) of participants had not used any contraceptive method, while 11.7% (N = 7) reported using oral contraceptive pills (OCPs). This trend may be attributed to the inclusion criteria, which focused on postpartum women. In the Saudi context, many women tend to breastfeed and may choose not to use contraception, relying on the lactational amenorrhea method instead. Given this context, lactational amenorrhea may be the most suitable method for this group, as it is scientifically proven to be effective for breastfeeding women for up to six months.

Participants’ knowledge of contraception

Table 2 illustrates that the mean score of participants’ knowledge about all methods of contraception significantly increased immediately following the educational intervention and remained elevated one month later compared to their mean knowledge prior to the intervention.

**Table 2:** Distribution of the study participants according to the mean level of knowledge about contraceptives before and after the educational session IUD, intrauterine device

Knowledge domain	Before educational session		Immediately after the educational session		After one month	
	Mean	SD	Mean	SD	Mean	SD
Standard days’ method	1.8	2.09	5.01	1.53	4.31	2.4
Symptoms-based fertility awareness method	1.05	1.74	4.78	1.81	3.73	2.64
Male condom	3.38	1.96	5.58	0.86	5.61	0.78
Female condom	0.78	1.68	5.35	1.38	4.95	1.83
Vaginal diaphragm	0.06	0.4	4.43	1.07	3.5	1.7
Cervical cap	0.05	0.38	4.06	1.38	2.96	1.89
Combined contraceptive pills	1.61	2.25	6.41	1.09	6.16	1.74
Progestin pills	0.56	1.69	6.16	1.13	6.3	1.74
Combined contraceptive injections	0.28	1.07	5.36	1.31	4.28	2.5
Progestin injections	0.18	0.89	4.91	1.86	4.21	2.49
Combined vaginal ring	0.41	1.06	6.01	1.56	5.26	2.47
Combined contraceptive patch	1.58	1.99	6.28	1.22	6.11	1.4
Implant (progestin under the skin)	1.41	2.05	5.13	1.47	4.58	2.14
Hormonal IUD	2.53	2.27	4.56	0.96	4.36	1.38
Emergency contraceptive methods	0.73	1.36	4.7	0.78	4.5	1.21
Permanent surgical methods	0.53	1.24	5.58	1.15	4.5	2.52

Table 3 demonstrates a significant improvement in participants’ knowledge from before to after the educational session. Prior to the intervention, 53 participants (88.3%) exhibited poor knowledge, while only seven (11.7%) had an average level of understanding. Immediately following the educational session, 55 participants (91.7%) displayed good knowledge. One month later, 46 participants (76.7%) retained good knowledge, and 15% had an average level of knowledge.

**Table 3:** Participants’ knowledge levels before and after the educational session

Level of knowledge	Before, N (%)	After, N (%)	After one month, N (%)
Poor (<40%)	53 (88.3)	1 (1.6)	5 (8.3)
Average (40-60%)	7 (11.7)	4 (6.7)	9 (15.0)
Good (>60%)	0 (0.0)	55 (91.7)	46 (76.7)

Furthermore, the study results indicate that the most prevalent source of information was relatives and friends (N = 41, 68.3%), followed by health workers (N = 16, 26.7%). Booklets and flyers were the least common sources of information (N = 4, 6.7%).

Participants’ intentions regarding contraceptive use and selected methods

Table 4 demonstrates that the educational session utilizing the booklet significantly enhanced participants’ intent to use contraceptives. The mean intent score after the session was markedly higher than before (p = 0.00).

**Table 4:** Differences in participants’ intent to use contraceptives before and after the educational session

Intent to use contraceptives	N	Mean difference	SD difference	t(df)	p-value
Before–after	60	-2.26	4.02	-4.358 (59)	0.000
Before–after one month	60	-1.66	5.43	-2.374 (59)	0.000

One month after the educational session, participants most commonly chose OCPs (N = 15, 25%), followed by the intrauterine device (IUD) and contraceptive implant (N = 13, 21.7%). Additionally, five participants (8.3%) selected patches, and one (1.7%) chose vaginal rings. The most common reason for choosing a contraceptive method before and after the educational session was “easy to use” (N = 11, 18.3% before), (N = 25, 41.7% after), and (N = 33, 55.0% after one month). The next most cited reason was “effective as a contraceptive method” (N = 10, 16.7% before), (N = 20, 33.3% immediately after), and (N = 30, 50.0% after one month).

Client feedback is a key indicator of service quality at any healthcare facility. We assessed participants’ feedback on the educational booklet. All participants (100%) reported reading the booklet, finding it useful, and recommending it to others. Moreover, 40 participants (66.7%) shared the booklet with others, such as their husbands, relatives, and friends.

## Discussion

The Middle East has recently experienced dramatic shifts in its population’s sociodemographic characteristics [10]. These changes have primarily impacted women, many of whom have attained higher education levels and entered the workforce. As a result, attitudes and behaviors toward fertility have evolved, leading to a greater adoption of contraceptive methods among women [10].

Our results indicate that over one-third of the participants were aged 25 to under 30 years, with more than half identifying as homemakers and the majority holding university degrees. The study further revealed that women employed outside the home and those with university degrees had significantly better knowledge of barrier methods compared to homemakers or those with only a secondary school education. Additionally, women living in urban areas demonstrated greater knowledge of natural and barrier methods than their rural counterparts.

These findings are consistent with a study assessing postpartum family planning knowledge, which found that women with higher educational levels had a better understanding of contraception [11]. Similarly, researchers in an Indian study noted that urban residents exhibited higher average knowledge of natural methods and condoms compared to those living in rural areas [12].

Regarding knowledge of contraceptive types, mechanisms of action, advantages and disadvantages, usage, and effectiveness, our study found that most participants had limited knowledge of various contraceptive methods prior to the educational session, except for male condoms. Participants’ mean knowledge of natural (fertility awareness-based) methods was particularly low, aligning with the findings of Alhusain et al. [7], who noted a limited understanding of the symptothermal method in a study conducted in Jeddah.

In terms of barrier methods, participants demonstrated adequate knowledge of male condoms but lacked understanding of female condoms, vaginal diaphragms, and cervical caps. This finding aligns with a Jeddah study, which similarly found that participants were well-informed about male condoms but not about other barrier methods [13]. In contrast, a study conducted in Tanzania among female undergraduates indicated better awareness of condoms and contraceptives [14], which may reflect differences in the educational background of participants, as all participants in the Tanzanian study were university students.

Prior to the educational session, participants also exhibited low knowledge of hormonal contraceptives. This finding is consistent with a 2017 study in Jeddah that highlighted poor knowledge of hormonal contraceptives before counseling [13]. Similarly, knowledge of emergency contraception was lacking before the educational session, reflecting results from a Saudi study that reported low awareness of this topic [15]. These findings echo trends seen in other Arab countries [16,17], suggesting a general lack of open discussion and effective counseling on emergency contraception.

Knowledge of permanent surgical methods of contraception was also low prior to the educational session, mirroring results from a study in the Hail region [18]. However, researchers in Uganda found that women aged 15-49 had good knowledge of permanent methods [19]. This discrepancy may be attributed to the fact that 60% of participants in the Ugandan study had received family planning information from clinic providers, highlighting variations in healthcare systems.

Following the educational session utilizing the booklet, 91.7% of participants demonstrated good knowledge, which remained high (76.7%) one month later. Knowledge of barrier methods significantly improved, supported by findings from a study indicating that 60% of participants showed increased knowledge of condoms after counseling [20].

Moreover, participants’ knowledge of hormonal methods improved following the educational session. This aligns with a randomized controlled trial in Jordan, where education using written materials enhanced women’s understanding of OCPs, with knowledge remaining relatively sustained even after a three-month follow-up [21]. These results emphasize the crucial role of education in enhancing contraceptive knowledge, indicating that women are eager to learn about contraceptive options to improve their well-being.

Knowledge of emergency contraception also significantly improved after the educational session, echoing findings from a quasi-experimental study in the United States, which demonstrated that video counseling effectively increased participants’ knowledge of all emergency contraception methods [22]. Our study also revealed a good mean knowledge score of participants regarding permanent surgical contraceptives after the educational session. Similarly, a quasi-experimental study in Jeddah found significant improvements in women’s knowledge of permanent surgical methods following counseling [13]. Conversely, a Pakistani study indicated that most participants had incomplete knowledge of bilateral tubal ligation, likely due to inadequate contraceptive counseling [23].

Understanding contraception is essential for making informed decisions about contraceptive methods [24]. Our study found that relatives and friends were the most common sources of contraceptive knowledge, followed by healthcare workers. This finding aligns with results from Karim et al., who noted that friends and family members serve as primary sources of information about emergency contraception [15].

The reliance on relatives and friends for contraceptive knowledge can be attributed to cultural norms in Saudi Arabia. In Saudi culture, postpartum women typically remain at home during the puerperal period, during which friends and relatives often visit, sharing their own experiences and information about contraceptives.

In our study, the most commonly used contraceptive method prior to the educational session was OCPs (11.7%), followed by male condoms (10.0%). In contrast, a broader study across various regions of Saudi Arabia found that 68.8% of women utilized contraception, with the most prevalent methods being OCPs, IUDs, coitus interruptus, and male condoms [25].

This discrepancy may arise from our focus on primiparae, whereas the referenced studies included women of all reproductive ages, regardless of parity status.

Our study’s results demonstrate that the educational session utilizing the booklet significantly increased participants’ intent to use contraceptives. The mean intent score after the educational session was markedly higher than before. This finding parallels a study conducted in the United States, which showed that contraceptive counseling during prenatal and postpartum periods greatly enhanced postpartum contraceptive use [26].

However, a contrasting study indicated that standardized postpartum contraceptive counseling did not influence adolescent mothers’ use of contraception [27]. This discrepancy may be attributed to differences in participants’ sociodemographic characteristics, particularly age and educational level. Our study included participants aged between 20 and 35 years, with 75% holding university degrees, while the American study focused on adolescents, 94% of whom had only a secondary school education or less.

Additionally, our study revealed that one month after the educational session, the most commonly chosen contraceptive methods were OCP, followed by IUDs and contraceptive implants. This result is consistent with two experimental studies conducted in Thailand and the United States, which found that introducing contraceptive education during the immediate postpartum period (i.e., before hospital discharge) significantly increased postpartum use of IUDs and contraceptive implants [28,29].

We also explored the reasons behind participants’ preferred contraceptive methods. The most frequently cited reasons for selecting a specific method before and after the educational session were “easy to use,” followed by “effective as a contraceptive method.” These findings are consistent with previous studies [30].

Strengths and limitations

Studies on the effect of contraceptive education on women’s knowledge and use of contraception are limited in some countries. Our novel research explored the effects of contraceptive counseling on the knowledge and use of contraception among primiparae in Saudi Arabia.

However, the study has several limitations that future researchers should address. First, the small sample size and the nonrandom sampling method in a single location (Jeddah) at one university hospital (KAUH) restrict the generalizability of our findings. Second, the pre-experimental design (one-group pretest-posttest) lacks the rigor of true experimental studies. Future research should adopt a randomized controlled trial design to more accurately assess cause-and-effect relationships and minimize bias.

## Conclusions

The educational session using an educational booklet significantly enhanced participants’ knowledge and intent to use modern contraceptives. Consequently, it is crucial for health authorities to prioritize the expansion of family planning services. Furthermore, assessing women’s knowledge levels and providing counseling by nurses is essential, as both nurses and physicians serve as primary information sources for patients.

Additionally, implementing an educational booklet on modern contraceptives in healthcare facilities would raise women’s awareness and facilitate the long-term dissemination of information. Future studies could benefit from a mixed research design that combines quantitative and qualitative methods to explore barriers to contraceptive use and the challenges associated with implementing contraceptive education programs in Saudi Arabian healthcare facilities.

## Appendices

Before the educational booklet

Social and demographic data:

Age

□ 20 > 25 Y             □ 25 > 30 Y           □ 30 > 35 Y            

Academic level

□ Capable of reading and writing   □ Intermediate □ secondary     

□ University

Employment

□ Housewife           □ employee          □ student

Husband academic level  

□ Capable of reading and writing   □ Intermediate □ Secondary     

□ University

Family monthly income

□ Less than 3000 SR    □ from 3000 to 6000 SR      □ more than 6000 SR

Place of residence

1.      وسائل منع الحمل التي استخدمتها سابقا ؟  (أشيري إلى جميع الوسائل اللتي استخدمتيها)

□ لا شيء                                           □حبوب منع الحمل الفمويه               □حقن منع الحمل

      □ اللصقه                                            □الحلقه  المهبليه   □           شريحة منع الحم   

□اللولب الهرموني               □الواقي الذكري □      غطاء عنق الرحم و مبيد النطاف

□الحاجز المهبلي ومبيد النطاف□  الوسائل المعتمده على الوعي بفترة الخصوبة     □ أخرى .......

Possibility to access أ the family planning methods

□ Yes                           □ No

Your information source regarding the latest contraceptive

o   Workers in the health field

o   Husband

o   Relatives and friends

o   Booklets and flyers

o   Never heard about them

o   Others….

**Table 5:** Assessment of modern contraception knowledge (before educational session)

Knowledge of the natural contraceptives (fertility awareness methods)					
	Method	Question	True	False	I don’t know
1	Do you know the standard days’ method? Yes No	1. Pregnancy occurs at the time of ovulation.			
		2. This method is based on determining the days of ovulation and then abstaining from sexual intercourse during the days of ovulation or using another contraceptive method such as the male or female condom.			
		3. This method is only used by women whose menstrual cycle ranges from 26 to 32 days.			
		4. The woman abstains from sexual intercourse from the eighth day of her period to the 19th day.			
		5. One of the advantages is that it has no side effects.			
		6. Cannot be used for those who have irregular menstrual cycles.			
2	Do you know a symptoms-based fertility awareness method? Yes No	1. Pregnancy occurs during the menstrual cycle.			
		2. The ovum life span is 24 hours and the sperm is 72 hours.			
		3. This method is based on determining ovulation days through cervical secretions and body temperature and then abstaining from sexual intercourse during these days or using another contraceptive method.			
		4. The symptoms that women know that she is in the fertile period and can get pregnant are transparent vaginal secretions that resemble egg white and the body temperature rises by half a degree.			
		5. The most important advantage is that it has no side effects.			
		6. One of its disadvantages is that it cannot be used for those who have vaginal infections.			
Knowledge of barrier methods					
3	Do you know the male condom? o Yes o No	1. The male condom is used to prevent the access of the sperm to the cervix.			
		2. The same male condom cannot be used more than once.			
		3. It is highly effective in contraception if used correctly.			
		4. One of the advantages of the male condom is that it protects against sexually transmitted diseases, including AIDS.			
		5. One of the disadvantages of the male condom is that it may cause allergy.			
		6. One of the disadvantages of the male condom is that it may slip or tear.			
4	Do you know the female condom? o Yes o No	1. The female condom is used to prevent the access of the sperm to the cervix.			
		2. The same female condom cannot be used more than once.			
		3. It is highly effective in contraception if used correctly.			
		4. One of the advantages of the female condom is that it protects against sexually transmitted diseases, including AIDS.			
		5. One of the disadvantages of the female condom is that it may cause allergy.			
		6. One of the disadvantages of the female condom is that it may slip or tear.			
5	Do you know the vaginal diaphragm? o Yes o No	1. For the first time using the vaginal diaphragm, it is necessary to consult the physician to determine the suitable size for the vagina.			
		2. It is placed inside the vagina before sexual intercourse and the vaginal diaphragm must be left in place for at least six hours after intercourse.			
		3. Vaginal diaphragm can be left inside the vagina for two or three days.			
		4. High effectiveness if it is used correctly.			
		5. One of its side effects is that it may cause irritation or itching to the vagina or penis.			
6	Do you know the cervical cap? o Yes o No	1. For the first time using the cervical cap, it is necessary to consult the physician to determine the suitable size for the cervix.			
		2. It is placed on the cervix before sexual intercourse and the cervical cap must be left in place for six hours after intercourse.			
		3. A cervical cap can be left in the cervix for two or three days.			
		4. High effectiveness if it is used correctly.			
		5. One of its side effects is that it may cause irritation or itching to the vagina or penis.			
Knowledge of hormonal methods					
7	Do you know the combined contraceptive pills? o Yes o No	1. It contains estrogen and progestin, and it prevents ovulation.			
		2. The start of using the contraceptive pills within the first five days of menstruation.			
		3. If the woman forgets one of the pills, she should take the pill as soon as she remembers and continue to take one pill on a daily basis after that.			
		4. High blood pressure, migraines, and deep vein thrombosis are conditions that prevent the use of combined contraceptive pills.			
		5. One of its advantages is that it is highly effective in contraception and reduces menstrual cramps and bleeding problems such as anemia.			
		6. One of its disadvantages is the change in the menstrual cycle such as interruption of the menstrual cycle or prolonged menstruation and irregularity.			
		7. Some antiepileptic, anti-depressants, and stress medications reduce their effectiveness.			
8	Do you know the progestin pills? o Yes o No	1. Progestin pills prevent ovulation and thus prevent pregnancy.			
		2. The start of using progestin pills during the first five days of menstruation.			
		3. Progestin pills are suitable for a breastfeeding woman and can be used after six weeks of childbirth.			
		4. If the woman forgets one of the pills, she should take it as soon as she remembers and continue to take one pill on a daily basis after that.			
		5. One of its advantages is that it is highly effective in contraception and reduces menstrual cramps and bleeding problems such as anemia.			
		6. One of its disadvantages is the change in the menstrual cycle such as interruption of the menstrual cycle or prolonged menstruation and irregularity.			
		7. Some antiepileptic, anti-depressants, and stress medications reduce their effectiveness.			
9	Do you know the combined contraceptive injections (on a monthly basis)? o Yes o No	1. Combined injections work to prevent ovulation and thus prevent pregnancy.			
		2. The woman begins using it during the first five days of the menstrual cycle, and then it is taken on the same date every month.			
		3. One of its advantages is that it is highly effective in contraception and reduces menstrual cramps and bleeding problems such as anemia.			
		4. One of its disadvantages is the change in the menstrual cycle such as interruption of the menstrual cycle or prolonged menstruation and irregularity.			
		5. Some antiepileptic, anti-depressants, and stress medications reduce their effectiveness.			
		6. Combined contraceptive injections are not used for women who suffer from deep vein thrombosis or breast cancer.			
10	Do you know the progestin injections? o Yes o No	1. The progestin injections work to prevent ovulation and thus prevent pregnancy.			
		2. It is suitable for breastfeeding women and can be used after six weeks of birth.			
		3. One of its advantages is that it is highly effective in contraception and reduces menstrual cramps and bleeding problems such as anemia.			
		4. One of its disadvantages is that the chances of childbearing are delayed for four months after stopping using them, and the bones may weaken.			
		5. Some antiepileptic, anti-depressants, and stress medications reduce their effectiveness.			
		6. Progestin injection is not used for women who suffer from deep vein thrombosis or breast cancer.			
11	Do you know the combined vaginal ring? o Yes o No	1. The vaginal ring works to prevent ovulation and thus prevent pregnancy.			
		2. The woman begins using it during the first five days of her menstrual cycle.			
		3. The woman puts it in the vagina for three weeks, then removes it in the fourth week, and menstruation begins.			
		4. One of its advantages is that it is safe and highly effective in contraception and reduces menstrual cramps and bleeding problems such as anemia.			
		5. One of its disadvantages is the changes in the menstrual cycle, such as interruption of the menstrual cycle or prolonged menstruation and irregularity.			
		6. Some antiepileptic drugs and some antidepressants and stress medications may reduce their effectiveness.			
		7. The vaginal ring is not used for the woman who suffers from deep vein thrombosis or breast cancer.			
12	Do you know the combined contraceptive patch? o Yes o No	1. The contraceptive patch works to prevent ovulation and thus prevent pregnancy.			
		2. The woman begins using it during the first five days of her menstrual cycle.			
		3. The woman puts it on the back, thigh, or lower abdomen for a week, then replaces it with a new patch for the following week and continues like this for three weeks, then the fourth week is without a patch.			
		4. One of its advantages is that it is safe and highly effective in contraception and reduces menstrual cramps and bleeding problems such as anemia.			
		5. One of its disadvantages is the changes in the menstrual cycle, such as interruption of the menstrual cycle or prolonged menstruation and irregularity.			
		6. Some antiepileptic drugs and some antidepressants and stress medications may reduce their effectiveness.			
		7. The contraceptive patch is not used for woman who suffers from deep vein thrombosis or breast cancer.			
13	Do you know the implant (progestin under the skin)? o Yes o No	1. The implant works to prevent ovulation and thus contraception.			
		2. it is placed under the skin in the upper arm.			
		3. The woman begins using it during the first five days of her menstrual cycle.			
		4. It is suitable for breastfeeding women and can be used after six weeks of birth.			
		5. One of its advantages is that it is safe and highly effective in contraception for many years.			
		6. One of its disadvantages is the changes in the menstrual cycle, such as interruption of the menstrual cycle or prolonged menstruation and irregularity.			
		7. Some antiepileptic drugs and some antidepressants and stress medications may reduce their effectiveness.			
14	Do you know the Hormonal IUD? o Yes o No	1. The IUD is placed inside the uterus.			
		2. The IUD is inserted during the first seven days of menstruation.			
		3. After birth, it will be inserted after four weeks.			
		4. The IUD is not used for people suffering from pelvic infections or sexually transmitted diseases.			
		5. One of its advantages is that it is very effective in contraception for a period of up to five years, and fertility returns quickly after its removal.			
		6. One of its disadvantages is the changes in the menstrual cycle, such as interruption of the menstrual cycle or prolonged menstruation and irregularity.			
15	Do you know the emergency contraceptive methods)? o Yes o No	1. Emergency contraceptive methods include two types, the copper IUD and the emergency contraceptive pills.			
		2. Emergency contraceptive methods prevent pregnancy if they are used during the first five days of unprotected intercourse. When used early, they are more effective for contraception.			
		3. If a woman forgot three pills of contraceptive pills and then had intercourse, the woman should use the emergency contraceptive pills.			
		4. If the male or female condom tears during sexual intercourse, the emergency contraceptive pill should be used.			
		5. The emergency contraceptive pill does not cause any problems for pregnancy if it has occurred.			
16	Do you know the permanent surgical methods? o Yes o No	1. Cutting the seminal canal (vasectomy) for men is considered a permanent contraceptive method			
		2. Cutting of the tubes carrying the ovum (fallopian tubes) for women is considered a permanent contraceptive method.			
		3. Very high effectiveness in contraception.			
		4. They have no side effects.			
		5. One of their advantages is that they do not affect the sexual performance of men or the sexual pleasure of the spouses.			
		6. One of their disadvantages is that fertility never returns, and tubes cannot be returned to their normal state again.			

You and birth control

1. The contraceptive method that you previously used? (Refer to all the methods you used)

□ None                     □ oral contraceptive pills          □ contraceptive injections

□ Patch                    □ vaginal ring                            □ contraceptive implant

□ IUD                      □ male condom                         □ Cervical cap & spermicide

□ Vaginal diaphragm and spermicide       □ fertility awareness based methods               □ others……….

One answer for each question

2. Which contraceptive method have you chosen to start using now? 

□ I have not chosen yet              □ fertility awareness-based methods

□ oral contraceptive pills          □ Patch                    □ vaginal ring

□ Contraceptive injections        □ IUD                     □ contraceptive implant

□ female condom      □ Vaginal diaphragm and spermicide     □ Cervical cap & spermicide

□ Others,……..

3. If you chose a contraceptive method, why did you choose it? (You can choose more than one option)

□ husband consent on it      □ easy for use     □ less side effects    □ effective as a contraceptive method                  □ I did not choose yet

4. How sure are you that you will use the contraceptive method for the next year?

□ not sure at all      □ somewhat sure      □ sure             □ very sure

5. How important is using the contraceptive method for you now?

□ not important at all      □ somewhat important    □ important    □ very important

6. How important is using a contraceptive method when you don't have sexual intercourse very often?

□ not important at all      □ somewhat important    □ important    □ very important

7. Do you think you will get pregnant if you use contraceptive methods correctly?

□ absolutely yes        □ probably yes         □ probably no        □ absolutely no

8. Do you think that contraceptive method will cause you bad side effects?

□ absolutely yes      □ probably yes    □ probably no     □ absolutely no

9. Is your husband object using contraceptive method?

□ absolutely yes      □ probably yes    □ probably no        □ absolutely no

10. Do you think it is difficult using contraceptive methods correctly?

□ absolutely yes      □ probably yes     □ probably no     □ absolutely no

11. Can you abstain from having sex if you did not use the contraceptive method?

□ not sure at all          □ somewhat sure           □ sure           □ Very sure

12. How sure are you going to use the contraceptive method in the future?

□ not sure at all          □ somewhat sure           □ sure          □ Very sure

13. Are you sure you will abstain from sex if your husband refuses to use the contraceptive method?

□ not sure at all        □ somewhat sure          □ sure           □ very sure

14. Do you think that contraceptive methods will do more harm than good for you?

□ absolutely yes      □ probably yes          □ probably no      □ absolutely no

15. How happy would you feel if you got pregnant in the next year?

□ I won't be happy at all     □ I'll be somewhat happy    □ I'll be happy    □ I'll be very happy

__________________________________________________________________________________________________________________________________________

After the educational booklet

Possibility to access to the family planning methods

□ Yes                           □ No

Your information source regarding the latest contraceptive

o   Workers in the health field

o   Husband

o   Relatives and friends

o   Booklets and flyers

o   Never heard about them

o   Others

**Table 6:** Assessment of modern contraception knowledge (after educational session)

Knowledge of the natural contraceptives (fertility awareness methods)					
	Method	Question	True	False	I don’t know
1	Do you know the standard days’ method? Yes No	1. Pregnancy occurs at the time of ovulation.			
		2. This method is based on determining the days of ovulation and then abstaining from sexual intercourse during the days of ovulation or using another contraceptive method such as the male or female condom.			
		3. This method is only used by women whose menstrual cycle ranges from 26 to 32 days.			
		4. The woman abstains from sexual intercourse from the eighth day of her period to the 19th day.			
		5. One of the advantages is that it has no side effects.			
		6. Cannot be used for those who have irregular menstrual cycles.			
2	Do you know a symptoms-based fertility awareness method? Yes No	1. Pregnancy occurs during the menstrual cycle.			
		2. The ovum life span is 24 hours and the sperm is 72 hours.			
		3. This method is based on determining ovulation days through cervical secretions and body temperature and then abstaining from sexual intercourse during these days or using another contraceptive method.			
		4. The symptoms that women know that she is in the fertile period and can get pregnant are transparent vaginal secretions that resemble egg white and the body temperature rises by half a degree.			
		5. The most important advantage is that it has no side effects.			
		6. One of its disadvantages is that it cannot be used for those who have vaginal infections.			
Knowledge of barrier methods					
3	Do you know the male condom? o Yes o No	1. The male condom is used to prevent the access of the sperm to the cervix.			
		2. The same male condom cannot be used more than once.			
		3. It is highly effective in contraception if used correctly.			
		4. One of the advantages of the male condom is that it protects against sexually transmitted diseases, including AIDS.			
		5. One of the disadvantages of the male condom is that it may cause allergy.			
		6. One of the disadvantages of the male condom is that it may slip or tear.			
4	Do you know the female condom? o Yes o No	1. The female condom is used to prevent the access of the sperm to the cervix.			
		2. The same female condom cannot be used more than once.			
		3. It is highly effective in contraception if used correctly.			
		4. One of the advantages of the female condom is that it protects against sexually transmitted diseases, including AIDS.			
		5. One of the disadvantages of the female condom is that it may cause allergy.			
		6. One of the disadvantages of the female condom is that it may slip or tear.			
5	Do you know the vaginal diaphragm? o Yes o No	1. For the first time using the vaginal diaphragm, it is necessary to consult the physician to determine the suitable size for the vagina.			
		2. It is placed inside the vagina before sexual intercourse and the vaginal diaphragm must be left in place for at least six hours after intercourse.			
		3. Vaginal diaphragm can be left inside the vagina for two or three days.			
		4. High effectiveness if it is used correctly.			
		5. One of its side effects is that it may cause irritation or itching to the vagina or penis.			
6	Do you know the cervical cap? o Yes o No	1. For the first time using the cervical cap, it is necessary to consult the physician to determine the suitable size for the cervix.			
		2. It is placed on the cervix before sexual intercourse and the cervical cap must be left in place for six hours after intercourse.			
		3. A cervical cap can be left in the cervix for two or three days.			
		4. High effectiveness if it is used correctly.			
		5. One of its side effects is that it may cause irritation or itching to the vagina or penis.			
Knowledge of hormonal methods					
7	Do you know the combined contraceptive pills? o Yes o No	1. It contains estrogen and progestin, and it prevents ovulation.			
		2. The start of using the contraceptive pills within the first five days of menstruation.			
		3. If the woman forgets one of the pills, she should take the pill as soon as she remembers and continue to take one pill on a daily basis after that.			
		4. High blood pressure, migraines, and deep vein thrombosis are conditions that prevent the use of combined contraceptive pills.			
		5. One of its advantages is that it is highly effective in contraception and reduces menstrual cramps and bleeding problems such as anemia.			
		6. One of its disadvantages is the change in the menstrual cycle such as interruption of the menstrual cycle or prolonged menstruation and irregularity.			
		7. Some antiepileptic, anti-depressants, and stress medications reduce their effectiveness.			
8	Do you know the progestin pills? o Yes o No	1. Progestin pills prevent ovulation and thus prevent pregnancy.			
		2. The start of using progestin pills during the first five days of menstruation.			
		3. Progestin pills are suitable for a breastfeeding woman and can be used after six weeks of childbirth.			
		4. If the woman forgets one of the pills, she should take it as soon as she remembers and continue to take one pill on a daily basis after that.			
		5. One of its advantages is that it is highly effective in contraception and reduces menstrual cramps and bleeding problems such as anemia.			
		6. One of its disadvantages is the change in the menstrual cycle such as interruption of the menstrual cycle or prolonged menstruation and irregularity.			
		7. Some antiepileptic, anti-depressants, and stress medications reduce their effectiveness.			
9	Do you know the combined contraceptive injections (on a monthly basis)? o Yes o No	1. Combined injections work to prevent ovulation and thus prevent pregnancy.			
		2. The woman begins using it during the first five days of the menstrual cycle, and then it is taken on the same date every month.			
		3. One of its advantages is that it is highly effective in contraception and reduces menstrual cramps and bleeding problems such as anemia.			
		4. One of its disadvantages is the change in the menstrual cycle such as interruption of the menstrual cycle or prolonged menstruation and irregularity.			
		5. Some antiepileptic, anti-depressants, and stress medications reduce their effectiveness.			
		6. Combined contraceptive injections are not used for women who suffer from deep vein thrombosis or breast cancer.			
10	Do you know the progestin injections? o Yes o No	1. The progestin injections work to prevent ovulation and thus prevent pregnancy.			
		2. It is suitable for breastfeeding women and can be used after six weeks of birth.			
		3. One of its advantages is that it is highly effective in contraception and reduces menstrual cramps and bleeding problems such as anemia.			
		4. One of its disadvantages is that the chances of childbearing are delayed for four months after stopping using them, and the bones may weaken.			
		5. Some antiepileptic, anti-depressants, and stress medications reduce their effectiveness.			
		6. Progestin injection is not used for women who suffer from deep vein thrombosis or breast cancer.			
11	Do you know the combined vaginal ring? o Yes o No	1. The vaginal ring works to prevent ovulation and thus prevent pregnancy.			
		2. The woman begins using it during the first five days of her menstrual cycle.			
		3. The woman puts it in the vagina for three weeks, then removes it in the fourth week, and menstruation begins.			
		4. One of its advantages is that it is safe and highly effective in contraception and reduces menstrual cramps and bleeding problems such as anemia.			
		5. One of its disadvantages is the changes in the menstrual cycle, such as interruption of the menstrual cycle or prolonged menstruation and irregularity.			
		6. Some antiepileptic drugs and some antidepressants and stress medications may reduce their effectiveness.			
		7. The vaginal ring is not used for the woman who suffers from deep vein thrombosis or breast cancer.			
12	Do you know the combined contraceptive patch? o Yes o No	1. The contraceptive patch works to prevent ovulation and thus prevent pregnancy.			
		2. The woman begins using it during the first five days of her menstrual cycle.			
		3. The woman puts it on the back, thigh, or lower abdomen for a week, then replaces it with a new patch for the following week and continues like this for three weeks, then the fourth week is without a patch.			
		4. One of its advantages is that it is safe and highly effective in contraception and reduces menstrual cramps and bleeding problems such as anemia.			
		5. One of its disadvantages is the changes in the menstrual cycle, such as interruption of the menstrual cycle or prolonged menstruation and irregularity.			
		6. Some antiepileptic drugs and some antidepressants and stress medications may reduce their effectiveness.			
		7. The contraceptive patch is not used for woman who suffers from deep vein thrombosis or breast cancer.			
13	Do you know the implant (progestin under the skin)? o Yes o No	1. The implant works to prevent ovulation and thus contraception.			
		2. it is placed under the skin in the upper arm.			
		3. The woman begins using it during the first five days of her menstrual cycle.			
		4. It is suitable for breastfeeding women and can be used after six weeks of birth.			
		5. One of its advantages is that it is safe and highly effective in contraception for many years.			
		6. One of its disadvantages is the changes in the menstrual cycle, such as interruption of the menstrual cycle or prolonged menstruation and irregularity.			
		7. Some antiepileptic drugs and some antidepressants and stress medications may reduce their effectiveness.			
14	Do you know the Hormonal IUD? o Yes o No	1. The IUD is placed inside the uterus.			
		2. The IUD is inserted during the first seven days of menstruation.			
		3. After birth, it will be inserted after four weeks.			
		4. The IUD is not used for people suffering from pelvic infections or sexually transmitted diseases.			
		5. One of its advantages is that it is very effective in contraception for a period of up to five years, and fertility returns quickly after its removal.			
		6. One of its disadvantages is the changes in the menstrual cycle, such as interruption of the menstrual cycle or prolonged menstruation and irregularity.			
15	Do you know the emergency contraceptive methods)? o Yes o No	1. Emergency contraceptive methods include two types, the copper IUD and the emergency contraceptive pills.			
		2. Emergency contraceptive methods prevent pregnancy if they are used during the first five days of unprotected intercourse. When used early, they are more effective for contraception.			
		3. If a woman forgot three pills of contraceptive pills and then had intercourse, the woman should use the emergency contraceptive pills.			
		4. If the male or female condom tears during sexual intercourse, the emergency contraceptive pill should be used.			
		5. The emergency contraceptive pill does not cause any problems for pregnancy if it has occurred.			
16	Do you know the permanent surgical methods? o Yes o No	1. Cutting the seminal canal (vasectomy) for men is considered a permanent contraceptive method			
		2. Cutting of the tubes carrying the ovum (fallopian tubes) for women is considered a permanent contraceptive method.			
		3. Very high effectiveness in contraception.			
		4. They have no side effects.			
		5. One of their advantages is that they do not affect the sexual performance of men or the sexual pleasure of the spouses.			
		6. One of their disadvantages is that fertility never returns, and tubes cannot be returned to their normal state again.			

You and birth control

1. The contraceptive method that you previously used? (Refer to all the methods you used)

□ None                    □ oral contraceptive pills          □ contraceptive injections

□ Patch                    □ vaginal ring                            □ contraceptive implant

□ IUD                      □ male condom                         □ Cervical cap & spermicide

□ Vaginal diaphragm and spermicide       □ fertility awareness based methods               □ others……….

One answer for each question

2. Which contraceptive method have you chosen to start using after reading the booklet? 

□ I have not chosen yet              □ fertility awareness based methods

□ oral contraceptive pills          □ Patch                    □ vaginal ring

□ Contraceptive injections        □ IUD                     □ contraceptive implant

□ female condom      □ Vaginal diaphragm and spermicide     □ Cervical cap & spermicide

□ Others,……..

3. If you chose a contraceptive method, why did you choose it? (You can choose more than one option)

□ husband consent on it      □ easy for use     □ less side effects    □ effective as a contraceptive method                  □ I did not choose yet

4. How sure are you that you will use the contraceptive method for the next year?

□ not sure at all      □ somewhat sure      □ sure             □ very sure

5. How important is using the contraceptive method for you now?

□ not important at all      □ somewhat important    □ important    □ very important

6. How important is using a contraceptive method when you don't have sexual intercourse very often?

□ not important at all      □ somewhat important    □ important    □ very important

7. Do you think you will get pregnant if you use contraceptive methods correctly?

□ absolutely yes        □ probably yes         □ probably no        □ absolutely no

8. Do you think that contraceptive method will cause you bad side effects?

□ absolutely yes      □ probably yes    □ probably no     □ absolutely no

9. Is your husband object using contraceptive method?

□ absolutely yes      □ probably yes    □ probably no        □ absolutely no

10. Do you think it is difficult using contraceptive methods correctly?

□ absolutely yes      □ probably yes     □ probably no     □ absolutely no

11. Can you abstain from having sex if you did not use the contraceptive method?

□ not sure at all          □ somewhat sure           □ sure           □ Very sure

12. How sure are you going to use the contraceptive method in the future?

□ not sure at all          □ somewhat sure           □ sure          □ Very sure

13. Are you sure you will abstain from sex if your husband refuses to use the contraceptive method?

□ not sure at all        □ somewhat sure          □ sure           □ very sure

14. Do you think that contraceptive methods will do more harm than good for you?

□ absolutely yes      □ probably yes          □ probably no      □ absolutely no

15. How happy would you feel if you got pregnant in the next year?

□ I won't be happy at all     □ I'll be somewhat happy    □ I'll be happy    □ I'll be very happy

Participants' feedback about the educational booklet

1- Have you read the educational booklet?         □ Yes           □ No

2- Was this booklet useful to you?                      □ Yes           □ No

3- Do you recommend the use of the booklet?    □ Yes           □ No

4- Did you share the educational booklet with others (your husband, relatives, friends?                                                           □ Yes           □ No

__________________________________________________________________________________________________________________________________________

تأثير الكتيب التعليمي المتعلق بوسائل منع الحمل الحديثة على المعرفة و العزم على الإستخدام لدى البكريات في

التقييم القبلي )

البيانات الاجتماعية و الديمغرافيي:

العمر

□ 20 < 25 سنه                 □ 25 < 30  سنه         □     30 < 35 سنه

المستوى التعليمي

□قادرة على القراءة و الكتابة    □متوسط          □ثانوي             □ جامعي

الوظيفة

□ ربة منزل                               □ موظفة                                   □  طالبة

المستوى التعليمي للزوج

□ قادر على القراءة والكتابة        □ متوسط     □ ثانوي               □جامعي

دخل الأسرة الشهري

□أقل من 3000ريال  □      من 3000 إلى 6000ريال             □أكثر من 6000 ريال

مكان الإقامة

□مدينه                   □قريه

إمكانية الوصول إلى وسائل تنظيم الأسرة

□نعم                     □لا

مصدر معلوماتك عن وسائل منع الحمل الحديثه:

      العاملين في مجال الصحة

      الزوج

      الأقارب والأصدقاء

      الكتيبات و المطويات

      لم أسمع بها

      أخرى ....................

**Table 7:** للتقييم المعرفة بوسائل منع الحمل الحديثة

	الوسيلة	السؤال	صح	خطأ	لا أعلم
1	هل تعرفين طريقة الأيام القياسية؟ نعم ( ) لا ( )	يحدث الحمل وقت التبويض			
		تعتمد هذه الطريقة على تحديد أيام التبويض ومن ثم الأمتناع عن الجماع خلال أيام التبويض أو استخدام وسيلة منع حمل أخرى كالواقي الذكري أو الأنثوي			
		هذه الطريقه تستخدمها فقط السيدة التي دورتها الشهرية من 26 إلى 32 يوم			
		تمتنع السيدة عن الجماع في الأيام من ثامن يوم من نزول الدوره إلى التاسع عشر			
		من مزاياها, ليس لها أعراض جانبية			
		لا يمكن استخدامها لمن لديها دورة غير منتظمه			
2	هل تعرفين وسيلة الوعي بالخصوبة المعتمدة على الأعراض؟ نعم ( ) لا ( )	يحدث الحمل أثناء الدورة الشهرية			
		تبلغ مدة حياة البويضه 24 ساعه و الحيوانات المنويه 72 ساعه.			
		تعتمد هذه الطريقة على تحديد أيام التبويض من خلال افرازات عنق الرحم ودرجة حرارة الجسم , ومن ثم الأمتناع عن الجماع خلال هذه الأيام أو استخدام وسيلة منع حمل أخرى			
		الأعراض التي تعرف بها المرأه أنها بفترة الخصوبة وقابلة للحمل هي افرازات المهبل الشفافه التي تشبه زلال البيض و ارتفاع درجة حرارة الجسم بمقدار نصف درجه			
		أهم مميزاتها ليس لها أعراض جانبية			
		من عيوبها صعوبة استخدامها لمن لديها التهابات مهبلية			
المعرفة بوسائل الحاجز					
3	هل تعرفين الواقي الذكري؟ نعم ( ) لا ( )	يستخدم الواقي الذكري لمنع وصول الحيوانات المنوية إلى عنق الرحم			
		لا يمكن استخدام نفس الواقي أكثر من مره			
		ذا فعالية عالية في منع الحمل إذا استخدم بطريقة صحيحة			
		من مميزات الواقي الذكري أنه يحمي من الأمراض المنقولة جنسيا بما فيها الأيدز			
		من عيوب الواقي الذكري أنه قد يسبب حساسية			
		من عيوب الواقي الذكري قد ينزلق أو يتمزق			
4	هل تعرفين الواقي الأنثوي؟ نعم ( ) لا ( )	يستخدم الواقي الأنثوي لمنع وصول الحيوانات المنوية إلى داخل المهبل			
		لا يمكن استخدام نفس الواقي أكثر من مره			
		ذا فعالية عالية في منع الحمل إذا استخدم بطريقة صحيحة			
		من مميزات الواقي الأنثوي أنه يحمي من الأمراض المنقولة جنسيا بما فيها الأيدز			
		من عيوب الواقي الأنثوي أنه قد يسبب حساسية			
		من عيوب الواقي الأنثوي قد ينزلق أو يتمزق			
5	هل تعرفين الحاجز المهبلي نعم ( ) لا ( )	في المرة الأولى لإستخدام الحاجز المهبلي, يجب مراجعة الطبيب لتحديد الحجم المناسب للمهبل			
		يوضع داخل المهبل قبل الجماع ويجب ترك الحاجز المهبلي في موضعه لمدة 6 ساعات بعد الجماع			
		يمكن ترك الحاجز المهبلي في المهبل لمدة يومين أو ثلاثة			
		فعاليه عاليه إذا استخدم بشكل صحيح			
		من أعراضه الجانبيه أنه قد يسبب تهيج و حكه للمهبل أو القضيب			
6	هل تعرفين غطاء عنق الرحم ؟ نعم ( ) لا ( )	في المرة الأولى لإستخدام غطاء عنق الرحم, لابد من مراجعة الطبيب لتحديد الحجم المناسب لعنق الرحم			
		يوضع قبل الجماع ويجب ترك غطاء عنق الرحم في موضعه لمدة 6 ساعات بعد الجماع			
		يمكن ترك الغطاء على عنق الرحم لمدة يومين أو ثلاثة			
		فعاليه عاليه إذا استخدم بشكل صحيح			
		من أعراضه الجانبيه أنه قد يسبب تهيج و حكه للمهبل أو القضيب			
المعرفة بالوسائل الهرمونيه					
7	هل تعرفين حبوب منع الحمل المركبة؟ نعم ( ) لا ( )	تحتوي على هرمون الإستروجين و البروجستين ,و تمنع التبويض			
		يكون البدء باستخدام الحبوب المركبة خلال الخمس أيام الأولى من الحيض			
		إذا نسيت السيدة أحد الأقراص , عليها أن تتناول الحبه فور تذكرها و تستمر في تناول قرص يوميا بعد ذلك			
		ارتفاع ضغط الدم و الصداع النصفي و الجلطات الوريدية العميقة هي حالات تمنع استخدام حبوب منع الحمل المركبة			
		من مميزاتها , أنها ذات فعالية عالية في منع الحمل و تقلل من تشنجات الحيض و مشاكل النزيف مثل فقر الدم			
		من عيوبها , تغيرات بالدورة الشهرية مثل إنقطاع الدورة أو طول مدة الحيض و عدم انتظامه			
		بعض أدوية الصرع و بعض مضادات الأكتئاب و التوتر تقلل من فعاليتها			
8	هل تعرفين حبوب “البروجستين فقط”؟ نعم ( ) لا ( )	تعمل حبوب البروجستين على منع التبويض و بالتالي منع الحمل			
		يكون البدء باستخدام حبوب البروجستين خلال الخمس أيام الأولى من الحيض			
		حبوب البروجستين تناسب السيدة المرضعة و تستطيع استخدامها بعد مرور 6 أسابيع من الولادة			
		إذا نسيت السيدة أحد الأقراص , عليها أن تتناول الحبه فور تذكرها و تستمر في تناول قرص يوميا بعد ذلك			
		من مميزاتها أنها ذات فعالية عالية في منع الحمل و تقلل من تشنجات الحيض و مشاكل النزيف مثل فقر الدم			
		من عيوبها تغيرات بالدورة الشهرية مثل إنقطاع الدورة أو طول مدة الحيض و عدم انتظامه			
		بعض أدوية الصرع و بعض مضادات الأكتئاب والتوتر تقلل من فعاليتها			
9	هل تعرفين حقن منع الحمل المركبة (الشهرية)؟ نعم ( ) لا ( )	تعمل الحقن المركبة على منع التبويض و بالتالي منع الحمل			
		تبدأ السيدة باستخدامها خلال الخمس أيام الأولى من الدورة الشهريه ثم تؤخذ بنفس الموعد كل شهر			
		من مميزاتها أنها ذات فعالية عالية في منع الحمل و تقلل من تشنجات الحيض و مشاكل النزيف مثل فقر الدم			
		من عيوبها تغيرات بالدورة الشهرية مثل إنقطاع الدورة أو طول مدة الحيض و عدم انتظامه			
		أدوية الصرع و بعض مضادات الأكتئاب والتوتر تقلل من فعاليتها			
		لا تستخدم الحقن المركبه لمن تعاني من جلطات الأوردة العميقة أو سرطان الثدي			
10	هل تعرفين حقن “البروجستين فقط”؟ نعم ( ) لا ( )	تعمل حقن البروجستين على منع التبويض و بالتالي منع الحمل			
		تناسب السيدة المرضعة و تستطيع استخدامها بعد مرور 6 أسابيع من الولادة			
		من مميزاتها أنها ذات فعالية عالية في منع الحمل و تقلل من تشنجات الحيض و مشاكل النزيف مثل فقر الدم			
		من عيوبها تأخر فرص الأنجاب لمدة 4 أشهر بعد التوقف عن استخدامها و قد تضعف العظام			
		بعض أدوية الصرع و بعض مضادات الأكتئاب والتوتر تقلل من فعاليتها			
		لا تستخدم حقن البروجستين لمن تعاني من جلطات الأوردة العميقة أو سرطان الثدي			
11	هل تعرفين الحلقة المهبلية المركبة؟ نعم ( ) لا ( )	تعمل الحلقة المهبلية المركبة على منع التبويض و بالتالي منع الحمل			
		تبدأ السيدة باستخدامها خلال الخمس أيام الأولى من الدورة الشهريه			
		تضعها السيدة في المهبل لمدة 3 أسابيع ثم تزيلها في الأسبوع الرابع وينزل الحيض			
		من مميزاتها أنها آمنه و ذات فعالية عالية في منع الحمل و تقلل من تشنجات الحيض و مشاكل النزيف مثل فقر الدم			
		من عيوبها تغيرات بالدورة الشهرية مثل إنقطاع الدورة أو طول مدة الحيض و عدم انتظامه			
		بعض أدوية الصرع و بعض مضادات الأكتئاب والتوتر تقلل من فعاليتها			
		لا تستخدم الحلقه المهبليه لمن تعاني من جلطات الأوردة العميقة أو سرطان الثدي			
12	هل تعرفين لصقة منع الحمل؟ نعم ( ) لا ( )	تعمل لصقة منع الحمل على منع التبويض و بالتالي منع الحمل			
		تبدأ السيدة باستخدامها خلال الخمس أيام الأولى من الدورة الشهريه			
		تضعها السيدة على الظهر أو الفخذ أو أسفل البطن لمدة أسبوع ثم تستبدلها بلصقة جديدة للأسبوع الذي يليه و تستمر هكذا لمدة ثلاث أسابيع , ثم يكون الأسبوع الرابع بدون لصقة			
		من مميزاتها أنها آمنه و ذات فعالية عالية في منع الحمل و تقلل من تشنجات الحيض و مشاكل النزيف مثل فقر الدم			
		من عيوبها تغيرات بالدورة الشهرية مثل إنقطاع الدورة أو طول مدة الحيض و عدم انتظامه			
		بعض أدوية الصرع و بعض مضادات الأكتئاب والتوتر تقلل من فعاليتها			
		لا تستخدم لصقة منع الحمل لمن تعاني من جلطات الأوردة العميقة أو سرطان الثدي			
13	هل تعرفين الشريحة (البروجيسترون تحت الجلد)؟ نعم ( ) لا ( )	تعمل على منع التبويض و بالتالي منع الحمل			
		توضع شريحة منع الحمل تحت الجلد في أعلى الذراع			
		تبدأ السيدة باستخدامها خلال الخمس أيام الأولى من الدورة الشهريه			
		تناسب السيدة المرضعة و تستطيع استخدامها بعد مرور 6 أسابيع من الولادة			
		من مميزاتها أنها آمنه و ذات فعالية عالية في منع الحمل على مدى سنوات			
		من عيوبها تغيرات بالدورة الشهرية مثل إنقطاع الدورة أو طول مدة الحيض و عدم انتظامه			
		بعض أدوية الصرع و بعض مضادات الأكتئاب والتوتر تقلل من فعاليتها			
14	هل تعرفين اللولب الهرموني؟ نعم ( ) لا ( )	يوضع اللولب داخل الرحم			
		يكون تركيب اللولب خلال أول سبع أيام من نزول الحيض			
		بعد الولادة: يكون تركيبه بعد 4 أسابيع			
		لا يستخدم اللولب لمن تعاني من التهابات الحوض أو الأمراض المنقولة جنسيا			
		من مميزاته انه ذو فعاليه عاليه جدا في منع الحمل و لمدة تصل إلى 5 سنوات وتعود الخصوبة سريعا بعد إزالته			
		من عيوبه , تغيرات بالدورة الشهرية مثل إنقطاع الدورة أو طول مدة الحيض و عدم انتظامه			
وسائل منع الحمل الطارئة					
15	هل تعرفين وسائل منع الحمل الطارئة؟ نعم ( ) لا ( )	وسائل منع الحمل الطارئة تشمل نوعين هما اللولب النحاسي و حبوب منع الحمل الطارئة			
		وسائل منع الحمل الطارئة تمنع حدوث الحمل إذا استخدمت خلال الخمس أيام الأولى من الجماع الغير محمي و عند استخدامها مبكرا تكون فعاليتها أكبر في منع الحمل			
		إذا نسيت السيدة 3 حبات من حبوب منع الحمل المركبة ثم حدث جماع , يجب على السيدة استخدام حبوب منع الحمل الطارئة			
		إذا تمزق الواقي الذكري أو الأنثوي أثناء الجماع , يجب استخدام حبوب منع الحمل الطارئة			
		حبوب منع الحمل الطارئة لا تسبب أي مشاكل للحمل اذا كان الحمل قد حدث فعلا			
وسائل منع الحمل الجراحية					
16	هل تعرفين الوسائل الجراحية الدائمة المفعول؟ نعم ( ) لا ( )	قطع القناه المنوية عند الرجل تعتبر من وسائل منع الحمل الدائمة			
		ربط أو قطع الأنابيب الناقلة للبويضة ( أنابيب فالوب) عند المرأة تعتبر من وسائل منع الحمل الدائمة			
		فعالية عاليه جدا في منع الحمل			
		من مميزاتها ليس لها أعراض جانبية			
		من مميزاتها لا تؤثر على الأداء الجنسي عند الرجل أو المتعه الجنسية عند الزوجين			
		من عيوبها لا تعود الخصوبة بعدها أبدا و لا يمكن إعادة الأنابيب إلى حالتها الطبيعية مرة أخرى			

أنت والحمل 

1.      وسائل منع الحمل التي استخدمتها سابقا ؟  (أشيري إلى جميع الوسائل اللتي استخدمتيها)

□ لا شيء                                           □حبوب منع الحمل الفمويه               □حقن منع الحمل

      □ اللصقه                                            □الحلقه  المهبليه   □           شريحة منع الحم   

□اللولب الهرموني               □الواقي الذكري □      غطاء عنق الرحم و مبيد النطاف

□الحاجز المهبلي ومبيد النطاف□  الوسائل المعتمده على الوعي بفترة الخصوبة     □ أخرى .......

أرجو اختيار إجابة واحدة لكل سؤال 

1.      ما هي وسيلة منع الحمل اللتي قد اخترت البدء في استخدامها حاليا؟

□أنا لم أختر بعد                        □      الوسائل المعتمده على الوعي بفترة الخصوبة    

□حبوب منع الحمل الفمويه         □       اللصقه                           □      الحلقة المهبلية

□حقن منع الحمل                            □ اللولب              □            شريحة منع الحمل

□ الواقي الأنثوي □       الحاجز المهبلي ومبيدات النطاف □   غطاء عنق الرحم ومبيد النطاف

□ أخرى تذكر .................

2.      إذا كنت قد اخترتي إحدى وسائل منع الحمل, لماذا اخترتيها؟ (يمكنك اختيار أكثر من خيار)

□موافقة الزوج عليها         □سهولة استخدامها       □أعراض جانبيه أقل      □فعال كمانع الحمل   □لم أختر بعد

3.      إلى أي حد أنت متأكده من أنك سوف تستخدمين وسيلة منع الحمل للعام المقبل؟

□لست متأكده على الإطلاق □         متأكده  إلى حد ما    □ متأكده              □ متأكده جدا

4.      ما مدى أهمية استخدام وسيلة منع الحمل الآن بالنسبة لك؟

□غير مهم على الإطلاق                        □ مهم نوعا ما     □             مهم         □  مهم جدا

5.      ما مدى أهمية استخدام وسائل منع الحمل عندما لا يكن لديك ممارسة الجنس في كثير من الأحيان؟

□غير مهم على الإطلاق         □ مهم نوعا ما      □ مهم   □     مهم جدا

6.      هل تعتقدين انك سوف تحملين إذا  استخدمت وسائل منع الحمل بشكل صحيح؟

□ بالتأكيد نعم     □  ربما نعم              □ على الأغلب لا     □  قطعا لا    

7.      هل تعتقدين أن وسائل منع الحمل سوف تسبب لك أعراض جانبية سيئة؟

□بالتأكيد نعم  □   ربما نعم            □ على الاغلب لا       □   قطعا لا

8.      هل زوجك ضد استخدامك لوسائل منع الحمل؟

□بالتأكيد نعم     □ ربما نعم              □ على الاغلب لا   □      قطعا لا     

9.      هل تعتقدين أنه من الصعب استخدام وسائل منع الحمل بشكل صحيح؟

□بالتأكيد نعم      □ ربما نعم            □ على الاغلب لا   □        قطعا لا    

10.   هل تستطيعين الامتناع عن  ممارسة الجنس في حال أنك لم تستخدمي وسيلة منع الحمل؟

□لست متأكدة على الإطلاق □ متأكدة إلى حد ما                  □  متأكدة               □ متأكدة جدا

11.   إلى أي حد  أنت متأكدة من أنك ستستخدمين وسيلة منع الحمل مستقبلا؟

□لست متأكدة على الإطلاق □  متأكدة إلى حد ما            □  متأكدة             □ متأكدة جدا

12.  هل  أنت متأكدة من أنك ستمتنعين عن ممارسة الجنس إذا كان زوجك يرفض استخدامك لوسائل منع الحمل؟

□لست متأكدة على الإطلاق   □  متأكدة إلى حد ما         □متأكدة                □ متأكدة جدا

13.  هل تعتقدين أن وسائل منع الحمل تضر أكثر مما تنفع بالنسبة لك؟

□ بالتأكيد نعم    □     ربما نعم       □على الاغلب لا              □    قطعا لا        

14.   ما مدى شعورك بالسعادة في حال حملك في العام المقبل؟

□لن أكون سعيدة على الإطلاق □    سأكون سعيدة إلى حد ما          □  سأكون سعيدة        □سأكون سعيدة جدا

______________________________________________________________________________________________________________________________

ملاحظتك حول الكتيب التعليمي ( بعدي فقط ) 

1.      هل قرأت الكتيب التعليمي؟                      □نعم       □                  لا

2.       هل كان الكتيب مفيد بالنسبة لك؟□      نعم      □                 لا

3.      هل توصين باستخدام الكتيب؟□        نعم                      □   لا

4.      هل شاركت الكتيب التعليمي مع الآخرين (زوجك, أقاربك, صديقاتك) ؟   □  نعم   □              لا
